# Role of *NPR1* in Systemic Acquired Stomatal Immunity

**DOI:** 10.3390/plants12112137

**Published:** 2023-05-29

**Authors:** Qijie Guan, Lisa David, Riley Moran, Ivan Grela, Angelica Ortega, Peter Scott, Lindsey Warnock, Sixue Chen

**Affiliations:** 1 Department of Biology, University of Mississippi, Oxford, MS 38677, USA; 2 Department of Biology, University of Florida, Gainesville, FL 32611, USA; 3 University of Florida Genetics Institue (UFGI), University of Florida, Gainesville, FL 32610, USA; 4 Plant Molecular and Cellular Biology Program, University of Florida, Gainesville, FL 32610, USA

**Keywords:** systemic acquired resistance (SAR), stomatal immunity, guard cell, pathogen defense, Non-expressor of Pathogenesis Related 1 (NPR1)

## Abstract

Stomatal immunity is the primary gate of the plant pathogen defense system. Non-expressor of Pathogenesis Related 1 (NPR1) is the salicylic acid (SA) receptor, which is critical for stomatal defense. SA induces stomatal closure, but the specific role of NPR1 in guard cells and its contribution to systemic acquired resistance (SAR) remain largely unknown. In this study, we compared the response to pathogen attack in wild-type Arabidopsis and the *npr1-1* knockout mutant in terms of stomatal movement and proteomic changes. We found that NPR1 does not regulate stomatal density, but the *npr1-1* mutant failed to close stomata when under pathogen attack, resulting in more pathogens entering the leaves. Moreover, the ROS levels in the *npr1-1* mutant were higher than in the wild type, and several proteins involved in carbon fixation, oxidative phosphorylation, glycolysis, and glutathione metabolism were differentially changed in abundance. Our findings suggest that mobile SAR signals alter stomatal immune response possibly by initiating ROS burst, and the *npr1-1* mutant has an alternative priming effect through translational regulation.

## 1. Introduction

Plant defense against pathogen attack relies on systemic acquired resistance (SAR), where long-distance mobile signals ‘prime’ the uninfected tissues and cells. SAR occurs when pathogens attack one part of the plant, and the plant responds by sending mobile signals from that primary site of infection throughout the vascular system to uninfected parts of the plant, allowing remote tissues to have a robust defense against pathogens [1,2,3,4,5]. SAR is known to depend on salicylic acid (SA) and its receptor, Non-expressor of Pathogenesis Related 1 (NPR1). The Arabidopsis *npr1* knockout mutant exhibits reduced transcription of a family of defense proteins called pathogenesis resistance (PR) proteins and becomes susceptible to disease [6]. SA is also required for stomatal defense, and SA synthesis mutants (*ics1* and *eds5/sid1/scord3*) and signaling mutant (*npr1-1*) are defective in stomatal defense [7].

The molecular functions and structure of the NPR1 protein have been unveiled. The N-terminus of NPR1 consists of a Broad-Complex, Tramtrack and Bric a brac (BTB) domain. This BTB domain interacts with NIMIN3 as part of SAR response, and the BTB domain may be an adapter for substrate ubiquitination by Cullin 3 E3 ligase, such as NPR3 and NPR4 with similar BTB domain structures, although NPR1 does not seem to interact directly with Cullin 3 [4]. The C-terminal domain of NPR1 contains a bipartite nuclear localization signal (NLS) and a putative transactivation domain. Upon SA induction, the NLS domain is required for translocation of NPR1 from its oxidized, oligomeric form in the cytosol, to the nucleus in its reduced, monomeric form [8,9]. Inside the nucleus, NPR1 interacts with TGA transcription factors through a domain of three or four ankyrin repeats. It has been proposed that the transactivation domain in the C-terminus of NPR1 allows it to serve as a coactivator of TGA transcription factors, ultimately leading to the increase in PR proteins during SAR response [6]. Interestingly, NPR1 from Arabidopsis has 17 cysteines, and 10 of those are highly conserved in the plant kingdom [4]. These cysteine residues make NPR1 redox-sensitive, and redox controls the activity of NPR1 during SAR [4]. SA regulates NPR1 by redox changes during SAR through mechanisms that are currently only partially understood [10]. Plant defense against pathogens requires the generation of reactive oxygen species (ROS), including superoxide, hydrogen peroxide (H_2_O_2_), and nitric oxide. During biotic stress, ROS are generated by either plasma-membrane localized NADPH/NADH oxidases (e.g., AtRBOHD and AtRBOHF), which generate superoxide that is converted to H_2_O_2_, or cell-wall-localized peroxidases that generate H_2_O_2_ into the apoplastic spaces [11,12].

Stomata are a first line of defense against pathogen entry, and they play an active role in limiting bacterial invasion as part of the plant innate immune system. This innate immune system is regulated by many factors, e.g., ROS [3,13,14]. A bacterial pathogen *Pseudomonas syringae* pv. Tomato (*Pst*) has evolved to counter stomatal defense, and it synthesizes a chemical called coronatine, a biochemical mimic of a plant hormone, jasmonic acid (JA)-isoleucine (Ile), that signals the guard cells to open stomata. The pathogen-associated molecular patterns (PAMP)-triggered stomatal immunity starts with the recognition of flagellin peptide by a FLAGELLIN SENSITIVE2 (FLS2) receptor kinase [15]. Through abscisic acid (ABA)-dependent and ABA-independent pathways, stomata close within an hour [3,7]. Plant hormones JA and SA have antagonistic relationships with each other and play important roles in pathogen defense [16]. Like JA, coronatine works in opposition to SA, which is involved in the closing of the stomata. Coronatine binds to the COI1 receptor of JA-Ile and causes stomata to reopen at 3 h after *Pst* infection [7,17]. A recent study reported that during stomatal reopening after 3 h exposure to *Pst* DC3000, a SMALL PHYTOCYTOKINES REGULATING DEFENSE AND WATER LOSS (SCREWs) triggers the PLANT SCREW UNRESPONSIVE RECEPTOR (NUT)-dependent phosphorylation of ABA INSENSITIVE1 (ABI1) and ABI2, which leads to an increase in the activity of ABI phosphatases towards OPEN STOMATA 1 (OST1), thereby promoting stomatal opening [18]. Coronatine does not affect stomatal closure by the SAR-related hormone SA [19]. As opposed to innate responses of guard cells to pathogen attack, SA signaling is a part of long-distance systemic pathogen response that initiates ROS burst via cell-wall-associated peroxidases [20].

We have previously found that SA is increased in the guard cells of primed Arabidopsis leaves [21,22]. We have also shown that Arabidopsis mutants lacking the Deficient in Induced Resistance 1 (DIR1) lipid transfer protein have decreased SA in primed guard cells and maintain a wider stomatal aperture when exposed to the *Pst* [23]. As a key protein for SAR, NPR1 as a receptor of SA is found in stomatal guard cells [8,9,10,24]. We hypothesized that NPR1 plays an important role in stomatal movement. The guard-cell-specific role of NPR1 could involve the regulation of the stomatal aperture in response to bacterial pathogens. Additionally, the loss of NPR1 could lead to changes in stomatal density or development, partially explaining the increased susceptibility of the *npr1* mutant. Guard cells primed for SAR could override the coronatine signal if they maintained increased levels of SA and ROS.

Here, we investigated the differences in stomatal movement between wild-type (WT) and *npr1-1* mutants to the bacterial pathogen *Pst* DC3000 under primed (SAR) or mock-treated conditions. We found that the *npr1-1* mutant had no significant difference in stomatal density during vegetative development compared to WT. Priming led to reduced stomatal apertures in WT Arabidopsis but not in the *npr1-1* mutant. Bacterial entry experiments of primed plants demonstrated that *npr1-1* systemic leaves were more susceptible to bacterial colonization compared to WT, suggesting stomatal aperture affects bacterial entry. Our results indicate that mobile SAR signals alter stomatal aperture, possibly by initiating ROS bursts in the apoplastic space surrounding guard cells. Proteomic analyses revealed molecular differences between the genotypic difference, priming effects, and *Pst* DC3000 response of WT and *npr1-1* systemic leaves. Although the *npr1-1* mutant does not have SAR activation with priming, we discovered that ribosomal proteins were overly abundant after *Pst* infection in *npr1*-primed leaves. The *npr1-1* mutant plants are deficient in immune response, making *npr1-1* more susceptible to pathogen attack. Elucidating guard-cell-specific responses to SAR is important for the development of transgenic crops that have enhanced pathogen defense without negative effects on growth and development.

## 2. Results

### 2.1. Bacterial Colonization of SAR Mutants

**Figure 1:**
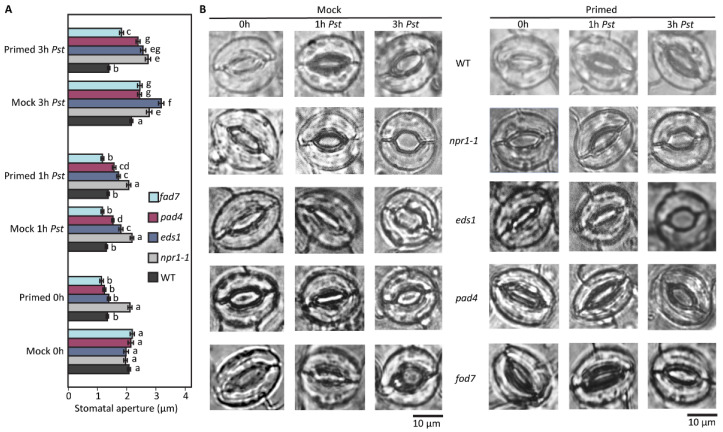
Differences in bacteria-caused stomatal movement phenotype among four SAR knockout mutants and the wild type, with and without pre-treatment (Mock and Primed). (**A**) Stomatal aperture changes in five genotypes of Arabidopsis with mock and primed pre-treatment under *Pst* DC3000 infection. Different letters (a–g) indicate significant differences. The significance was calculated by a one-way ANOVA test with a significance level of 0.05. (**B**) Representative microscopic images of stomatal guard cells during the exposure period of *Pst* DC3000.

We analyzed SAR-related mutants to compare stomatal responses to priming and to *Pst* exposure (Figure 1). SA function involves flavin-dependent monooxygenase 1 (FMO1), enhanced disease susceptibility 1 (EDS1), and phytoalexin-deficient 4 (PAD4), which are the other components of SAR [25]. EDS1 and PAD4 are lipase-like proteins that are both required for the accumulation of SA during SAR. Direct interaction between PAD4 and EDS1 has been confirmed by yeast-two-hybrid and coimmunoprecipitation experiments [25]. Fatty acid desaturase 7 (FAD7) is a chloroplastic enzyme involved in the production of 16:3 and 18:3 fatty acids from galactolipids, sulpholipids, and phosphatidylglycerol, and it is induced by wounding independent of JA in shoots. Out of the mutants, we found that *npr1-1* was unique in its response to priming at 0 h, as it was the only SAR mutant to have an open stomatal aperture after priming. All other SAR mutants *eds1*, *pad4* and *fad7* had stomata with narrow apertures that were not significantly different from WT after priming (Figure 1). However, response to *Pst* exposure at 1 h varied with the different mutants. Although *npr1-1* did not close stomata in response to *Pst* in either primed or mock plants, the *eds1* and *pad4* also had larger stomata apertures than WT, but they were narrower than *npr1-1* at 1 h after *Pst* exposure in both mock and primed plants. After 3 h exposure to *Pst,* the coronatine from the *Pst* also had different effects on the SAR mutants. Compared to WT, all the SAR mutants had larger stomata apertures in the mock and primed plants. However, compared to *npr1-1*, only *eds1* had a larger stomatal aperture at 3 h in the mock plants. In the primed plants, all the SAR mutants had wider stomatal apertures than WT, with *npr1-1* having the widest stomatal aperture at 3 h after *Pst* exposure in the primed plants (Figure 1). In this study, we chose to focus on studying *npr1-1*.

### 2.2. Stomatal Density in the Course of Vegetative Development of Wild Type and npr1-1 Mutant

**Figure 2:**
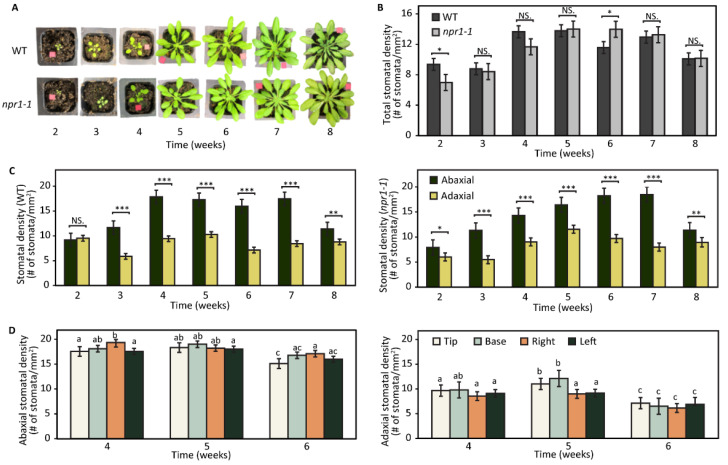
Phenotype and stomatal density during vegetative development of wild-type and *npr1-1* mutant. (**A**) Growing and leaf area changes during the 8 weeks of the vegetative development stage. (**B**) Total stomatal density of wild-type and *npr1-1* mutant Arabidopsis leaves during the vegetative development stage. (**C**) Abaxial and adaxial stomatal density of wild-type and *npr1-1* mutant Arabidopsis leaves during the vegetative development stage. (**D**) Different quadrants of abaxial and adaxial stomatal density of the *npr1-1* mutant Arabidopsis leaves in 4–6 weeks of the vegetative development stage. Different letters (a–c) indicate significant differences. The significance was calculated using a one-way ANOVA test with a significance level of 0.05. *: *p*-value <= 0.05, **: *p*-value <= 0.01, ***: *p*-value <= 0.001, NS: not significant.

Arabidopsis WT and *npr1-1* mutant plants grown under short-day conditions exhibited little phenotypic differences up to eight weeks old (Figure 2A). They had similar overall stomatal density, with exceptions at weeks 2 and 6 (Figure 2B). Stomatal density was consistently lower on the adaxial leaf surface compared to the abaxial from weeks 3–7, with no significant difference in weeks 2 and 8. Both WT and *npr1-1* exhibited an increase in stomatal density from weeks 2 to 5 (peaking at week 5), followed by a decline from weeks 6 to 8. Notably, the only discrepancy in total stomatal density between the *npr1-1* and WT plants occurred at week 6 (Figure 2C). This study is the first to compare stomatal density between the abaxial and adaxial leaf surfaces in Arabidopsis during vegetative development, with results indicating similar densities across different leaf quadrants, except for an increased stomatal density in the tip and base quadrants of the adaxial side at week 5 (Figure 2D).

### 2.3. Stomatal Movement of Wild Type and npr1-1 in Response to Pst DC3000

**Figure 3:**
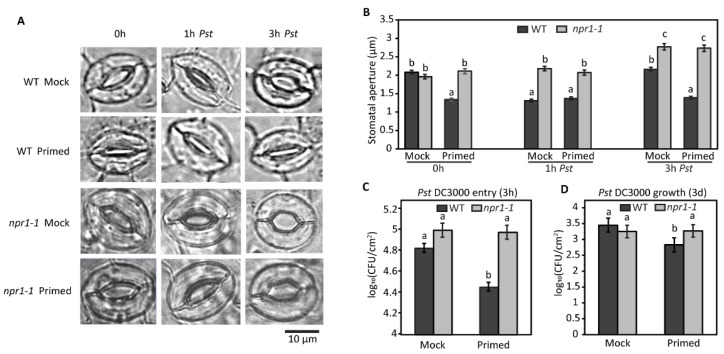
Stomatal movements and bacterial colonization in response to the priming of the wild type and *npr1-1*. (**A**) Representative microscope image of stomatal guard cells during the exposure period of *Pst* DC3000 in WT and *npr1-1* Arabidopsis with mock and primed pre-treatments. (**B**). Stomatal apertures during the exposure period of *Pst* DC3000 in WT and *npr1-1* Arabidopsis with mock and primed pre-treatments. (**C**) Bacterial entry result of mock and primed systemic leaves of the WT and *npr1-1* Arabidopsis after 3 h of *Pst* DC3000 infection. (**D**) Bacterial growth result of mock and primed systemic leaves of the WT and *npr1-1* Arabidopsis after 3 days of *Pst* DC3000 infection. Different letters (a–c) indicate significant differences. The significance was calculated using a one-way ANOVA test with a significance level of 0.05.

To determine stomatal aperture differences between WT and *npr1-1* plants, we injected either *Pst* (0.02 OD_600_) or 10 mM MgCl_2_ (mock) into one mature rosette leaf of 5-week-old plants with a needleless syringe. After 3 days, we exposed the opposite distal rosette leaf to the pathogen (0.2 OD_600_) and compared the response of the stomata in the primed *npr1-1* mutant and WT plants to each other and to the mock-treated plants. As previously reported [7,13], the basal immune response of the stomata is to close after 1 h of exposure to *Pst* and then reopen after 3 h of *Pst* treatment. This basal stomatal response occurred in the mock-infiltrated WT leaves, where the average stomatal aperture was approximately 2 µm at 0 h, decreased to approximately 1.3 µm after 1 h, and then increased to approximately 2 µm at 3 h after exposure to *Pst*. Surprisingly, the primed WT plants did not exhibit this typical stomatal immune response. The distal leaves from the primed WT plants had stomata with an average aperture of approximately 1.3 µm from 0 to 3 h after exposure to *Pst*. There was no significant difference in the stomatal aperture measurements from the primed leaves taken at 0, 1, and 3 h after exposure to the pathogen (Figure 3B). In contrast to the WT stomata response, the *npr1-1* mutant showed no significant difference in stomatal aperture between the mock and primed leaves at 0, 1, or 3 h after *Pst* exposure. The average stomatal aperture for 0 and 1 h after *Pst* exposure for the *npr1-1* mutant was approximately 2 µm for both the primed and mocked plants. However, at 3 h after *Pst* exposure, the stomata aperture of both the primed and mocked *npr1-1* plants was approximately 2.7 µm, significantly greater than either the *npr1-1* or WT mock or primed leaves at any of the other time points (Figure 3A,B).

### 2.4. Bacterial Colonization of Wild Type and npr1-1

To examine if the stomata apertures affected bacterial entry through the stomatal pores and into the apoplastic spaces of the leaves, we conducted bacterial entry assays for mock and primed systemic leaves of the WT and *npr1-1* mutant. After 3 h of exposure to *Pst,* 2.2 times more bacteria entered the apoplasts in the leaves of WT mock compared to the WT primed plants. In contrast, the *npr1-1* mocked and primed plants showed no significant difference in bacterial entry after 3 h of exposure to *Pst* (Figure 3C). Additionally, both *npr1-1* mock and primed leaves had significantly greater numbers of bacteria that entered the leaf apoplasts when compared to the WT primed plants (Figure 3C).

To examine susceptibility to *Pst,* we measured bacterial growth in the mocked and primed systemic leaves of the two genotypes. Approximately 5.4 times more bacteria colonized the apoplasts in the leaves of WT mock compared to the WT primed plants. In contrast, the *npr1-1* mock and primed plants showed no significant difference in bacterial growth after 3 days of exposure to *Pst*. Additionally, both *npr1-1* mocked and primed leaves had significantly greater numbers of bacteria colonizing the leaf apoplasts when compared to WT primed leaves (Figure 3D).

### 2.5. Redox Assays Revealed Decreased ROS and Lipid Peroxidation in Wild-Type Primed Leaves

**Figure 4:**
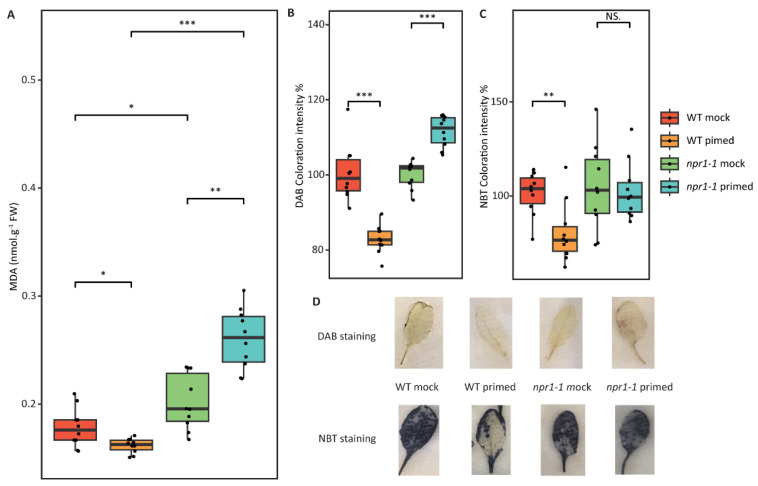
Lipid peroxidation and ROS analyses of WT and *npr1-1* Arabidopsis systemic leaves with mock and primed treatments before subsequent *Pst* exposure. (**A**) MDA levels of WT and *npr1-1* Arabidopsis systemic leaves with mock and primed treatment. (**B**) DAB concentration of WT and *npr1-1* Arabidopsis systemic leaves with mock and primed treatment. (**C**) NBT concentration of WT and *npr1-1* Arabidopsis systemic leaves with mock and primed treatment. (**D**) Representatives of DAB and NBT staining result of the four groups. *: *p*-value <= 0.05, **: *p*-value <= 0.01, ***: *p*-value <= 0.001, NS: not significant.

Here, lipid peroxidation and ROS levels were analyzed using malondialdehyde (MDA), DAB, and NBT staining. As shown in Figure 4, WT leaves that had been primed had lower levels of lipid peroxidation, hydrogen peroxide, and superoxide when compared to WT mock leaves. Although *npr1-1* mock leaves had no significant difference from WT mock leaves, there was a significant difference when comparing primed *npr1-1* to primed WT. Primed *npr1-1* leaves had 1.7 times higher levels of MDA, indicating lipid peroxidation, when compared to primed WT or mocked WT or *npr1-1*. DAB and NBT assays also indicated slightly higher levels of hydrogen peroxide and superoxide, respectively, when comparing primed *npr1-1* to primed WT leaves (Figure 4).

### 2.6. Proteomic Differences of npr1-1 and Wild Type Arabidopsis

In our study, we identified 1785 proteins with a minimum of two unique peptides and a false discovery rate (FDR) cutoff of 1% (Table S1). Principle component analysis indicated that in the proteome, the WT mock group was different from the *npr1-1* mock group before subsequent *Pst* exposure, and WT and *npr1-1* showed very different proteomic changes after the subsequent *Pst* exposure (Figure S1). Thus, in this study, we focus on the genotypic differences and the proteomic changes after the subsequent *Pst* exposure.

Among the identified proteins, 452 exhibited differential abundance (*p*-value ≤ 0.05) between the *npr1-1* mock group and the WT mock (i.e., genotypic differences). Specifically, 301 proteins were significantly increased in the *npr1-1* mock group, while 151 proteins were significantly decreased. The fold changes in the differential proteins ranged from 1.1-fold to about 40-fold. By mapping these differentially abundant proteins (DAPs) to KEGG Arabidopsis pathways, we found these proteins are related to metabolic pathways (150/452), biosynthesis of secondary metabolites (84/452), carbon metabolism (39/452), and biosynthesis of amino acids (26/452). Interestingly, of the 20 most DAPs, 18 of them were decreased in the *npr1-1* mutant (Table 1).

### 2.7. Proteome Changes in npr1-1 and Wild Type after Pst DC3000 Infection

The response to *Pst* DC3000 infection varied among the WT mock, WT primed, *npr1-1* mock, and *npr1-1* primed groups. After 3 h of infection, the WT mock group had 467 DAPs, including 266 increased and 201 decreased proteins, and the WT primed group had 336 DAPs, including 228 increased and 102 decreased proteins. In the *npr1-1* mutant, the mock group had 337 DAPs after 3 h exposure to *Pst* DC3000, including 206 increased and 131 decreased proteins, while the primed group had 526 DAPs, including 262 increased and 264 decreased proteins.

There were 21 proteins that changed significantly after 3 h of pathogen exposure in all of the WT mock, WT primed, *npr1-1* mock and *npr1-1* primed groups (Table 2). Among them, 20 DAPs had the same changing patterns in all four groups except a CAX-interacting protein (CXIP2). CXIP2 increased in the WT mock and WT primed group but decreased in the *npr1-1* mock and *npr1-1* primed group.

By comparing the DAPs in the WT mock group and WT primed group after 3 h *Pst* DC3000 exposure, 335 DAPs were found to be unique in the WT mock group, 204 DAPs were unique in the WT primed group, and 132 DAPs were shared. When comparing the proteome profile between the WT mock group and the WT primed group after pathogen exposure, the DAPs were highly enriched in GO in terms of response to osmotic tress, response to temperature stimulus, oxidation-reduction process, photosynthesis, and nitrogen compound metabolic process. These GO terms are highly correlated to stomatal movement.

There were 337 DAPs in the *npr1-1* mock group and 526 DAPs in the *npr1-1* primed group after 3 h of *Pst* DC3000 infection, including 135 shared DAPs, 202 *npr1-1* mock-changed-only proteins and 391 *npr1-1* primed-changed-only proteins. Notably, the number of DAPs after 3 h of pathogen exposure in the *npr1-1* primed group exceeded that of the WT mock group. The 20 most changed proteins were selected to represent the changed abundance after *Pst* DC3000 infection in the *npr1-1* primed group only (Table 3). Of the 20 proteins, only three decreased in abundance after pathogen exposure. In the remaining 17 decreased proteins, 14 were ribosomal proteins, and their fold changes were greater than those of any other DAPs in the *npr1-1* mock group (with the highest log2FC being 2.44).

### 2.8. Glutathione Metabolism of npr1-1 and Wild Type Arabidopsis after Pst DC3000 Infection

**Figure 5:**
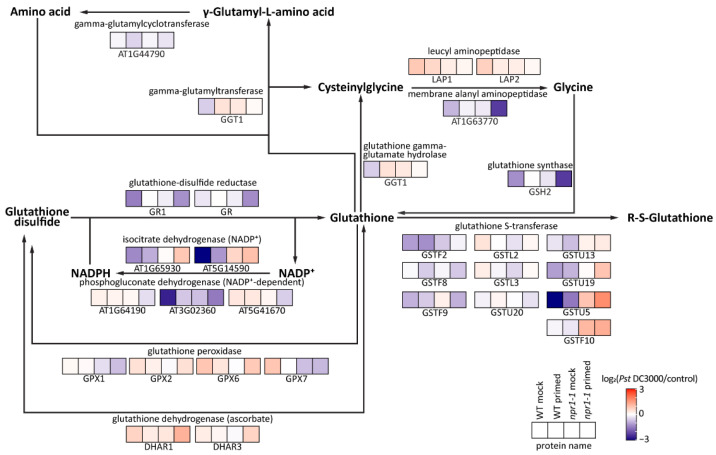
Protein abundance changes after 3 h of *Pst* DC3000 infection in the glutathione metabolism of the systemic leaves of WT mock, WT primed, *npr1-1* mock, and *npr1-1* primed Arabidopsis.

A total of 30 enzymes involved in glutathione metabolism were identified (Figure 5). Notably, the observed alterations in protein expression, encompassing glutathione S-transferase (GST), glutathione peroxidase, and isocitrate dehydrogenase, underscore the distinct ROS responses exhibited by the WT mock, WT primed, *npr1-1* mock, and *npr1-1* primed groups after *Pst* DC3000 infection. GSTU5, which was co-regulated with the glutathione reductase activities [26], increased most in the *npr1-1* primed group and decreased most in the WT mock group. The abundance of the isocitrate/isopropylmalate dehydrogenase (AT5G14590) decreased after *Pst* DC3000 infection in WT, and increased in *npr1-1* mutant, regardless of whether the systemic leaves were primed or not. Furthermore, it was observed that this protein exhibited a significant decrease in the *npr1-1* mutant compared to the WT (Table 1).

## 3. Discussion

### 3.1. NPR1 Does Not Regulate Stomatal Density

Guard cells respond to the perception of pathogens by altering stomatal apertures, an important basal defense response that limits bacterial entry into the apoplastic space of leaf tissues. We investigated the stomatal density of the *Pst*-susceptible *npr1-1* mutant to test the hypothesis that the increased susceptibility was due to the increased density of stomata, given that *Pst* uses stomata pores to access the apoplastic spaces of the leaf tissue for colonization. We found that stomatal density in the *npr1-1* mutant and WT were not significantly different during vegetative growth (Figure 2). Both WT and *npr1-1* had higher stomatal density on the abaxial compared to the adaxial sides of the leaves during weeks 3–7 of growth. However, at week 2 and week 8 of vegetative growth, the two sides of the leaves were mostly even in stomatal density. We also found that the abaxial sides of the WT leaves showed equal stomatal density across the leaf surface from weeks 4 to 6. However, the adaxial side decreased overall density at week 6 and decreased density in the center areas of the leaf at week 5. To our knowledge, stomatal density counts on different leaf surfaces and areas during the vegetative development of Arabidopsis have not been reported. Clearly, NPR1 does not appear to regulate stomatal density/developmental processes. Although we did not observe significant differences in stomatal density between the WT and *npr1-1*, we did observe interesting developmental differences between the abaxial and adaxial sides of the leaves.

### 3.2. Stomatal Reopening by SCREW-NUT Function Is NPR1-Independent

Despite the similar numbers of stomatal pores between the susceptible mutant *npr1-1* and the resistant WT, the mechanism of response of guard cells to the pathogen was different when we compared *npr1-1* to WT. We investigated the stomatal movement of the *npr1-1* mutant vs. the WT plants by measuring the stomatal aperture of distal leaves 72 h after one leaf was infiltrated with *Pst* (primed) vs. a mock solution (control). Surprisingly, we found that the WT distal leaves from primed plants had a much smaller stomata aperture when compared to the control plants. Interestingly, the stomata from primed leaves were able to maintain the same narrow aperture after 3 h of exposure to *Pst* (Figure 3). The stomata of control WT had a standard, well-documented response, in that they closed at 1 h after perception of *Pst* by pattern recognition receptors in the guard cells, then reopened at 3 h after *Pst* exposure due to the presence of the coronatine, the JA-Ile mimic produced by *Pst* [7,15]. The stomata of primed distal leaves of the *npr1-1* mutant did not have a narrowed aperture as the WT primed leaves did. Additionally, the stomatal aperture of the *npr1-1* primed and mock plants were the same at all the time points (0, 1, and 3 h). Interestingly, at 3 h after exposure to *Pst*, the *npr1-1* stomata of both the mock and primed plants had larger apertures than either WT mock or primed plants. The larger stomatal apertures of the *npr1-1* mutant corresponded to increased bacterial entry. On the other hand, the narrow stomatal apertures of the primed WT plants corresponded to lower numbers of bacteria that were able to enter the leaves (Figure 3). It has been reported that stomata reopen after 3 h exposure under *Pst* DC3000 SCREW-NUT-dependent phosphorylation of ABI1 and ABI2, which leads to an increase in the activity of ABI phosphatases towards OST1, a key kinase that mediates ABA- and PAMP-induced stomatal closure, and a reduction in the activity of S-type anion channels [18]. According to our phenotype result, *npr1-1* mutation prevented stomatal closure but not stomatal reopening. This result indicates that SCREW-NUT regulates stomatal reopening in an *NPR1*-independent manner.

### 3.3. Translation Regulation Is an Alternative Priming Effect of npr1-1 Knockout Arabidopsis

With PCA-based machine learning, the priming effect and the subsequent *Pst* DC3000 exposure separate well between the two genotypes (Figure S1). One protein that showed different and significant patterns in the two genotypes under 3 h of *Pst* infection was CXIP2. CXIP2 interacts with CAX1 [27], and disruption of CAX1 causes the activation of pathogen defense mechanisms, probably through the manipulation of calcium homeostasis [28]. It is reported that calcium-dependent protein kinase CPK5 contributes to priming, which could enhance basal resistance without the enhancement of SAR in an Arabidopsis *sard1-1* mutant [29]. CPK5 is involved in RBOHD-mediated ROS signaling [30], and glutathione can act as a key signaling factor through redox homeostasis [31].

Clearly, WT plants have an ROS response to systemic SAR signals that affect the basal responses of guard cells to pathogens that *npr1-1* mutant plants lack (Figure 4). We propose the ROS burst from priming that affects stomata aperture is mainly created by peroxidases via the SA pathway, not only by the NADPH oxidases via the ABA and flagellin pathway, as previously described [18]. It has been reported that coronatine inhibits stomatal closure by flg22, ABA, and darkness, which trigger ROS production through NADPH oxidases, particularly RBOHD and RBOHF, in Arabidopsis [15,19,32]. Stomatal closure triggered by SA relies on apoplastic peroxidase-dependent ROS production, and cell-wall-located enzymes have been proven to be responsible for the apoplastic ROS production, which is not affected by coronatine [33]. Controlling stomata aperture with a SA/peroxidase/SAR-like response can limit the negative effects on the growth and development of SAR because the narrow stomata of primed plants can reduce the number of bacteria that colonize the leaf.

An alpha/beta hydrolase superfamily protein (ABH) AT4G12830, is the most decreased protein in WT Arabidopsis after priming and is also the most decreased protein in *npr1-1* primed Arabidopsis after *Pst* DC3000 infection. ABHs are often linked to housekeeping roles, e.g., fundamental cellular functions involving the degradation and recycling of cellular metabolites, processing of extracellular nutrients, and detoxification of xenobiotics. [34]. ABHs support a variety of unique catalytic functions. For example, ABH superfamily protein SABP2 is a MeSA esterase, hydrolyzing MeSA to produce active SA, and participates in feedback inhibition to control the strength and duration of the SA response [34]. Although AT4G12830 is an uncharacterized ABH-like protein, it may be an important protein involved in Arabidopsis pathogen response, a hypothesis to be tested in due course.

**Figure 6:**
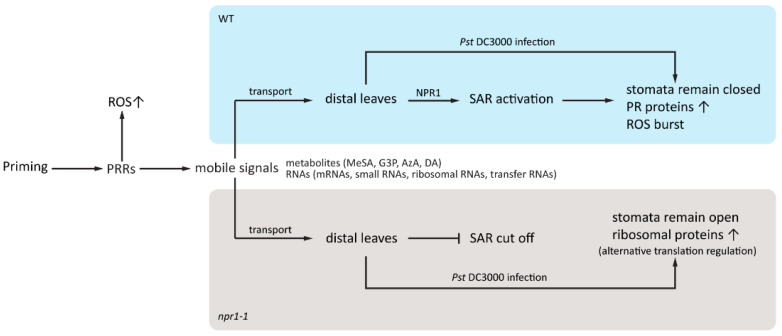
Schematic diagram showing priming effect on stomatal immunity in distal leaves and alternative translation regulation in *npr1-1* primed Arabidopsis. After priming, the leaf generates several mobile signals to distal leaves, including Methyl SA and ribosomal RNAs.

One interesting finding of our study is that several ribosomal proteins increased in *npr1-1* primed systemic leaves after *Pst* DC3000 infection. Importantly, prior to pathogen exposure, these ribosomal proteins did not show significant differences in abundance between the *npr1-1* mock and *npr1-1* primed groups, nor did they exhibit any significant differences in the WT primed groups after pathogen exposure. This suggests that these ribosomal proteins play important roles in pathogen defense due to the failure of being primed in the *npr1-1* plants. We further propose that translational regulation may serve as a bypass mechanism for plant response to priming in the absence of an effective SAR signaling pathway (in *npr1-1*), as indicated by significantly higher levels of MDA in *npr1-1* primed leaves compared to *npr1-1* mock leaves. Based on the proteomic results, the translational regulation seems to be selective in the protein profiles but ineffective in the output of stomatal immune response (Figure 6).

## 4. Materials and Methods

### 4.1. Plant Growth

The *Arabidopsis thaliana* wild-type Col-0 and *npr1-1* mutant seeds were provided by Dr. Zhonglin Mou at the University of Florida. The seeds were suspended in 20 mL of deionized H_2_O and vernalized in the refrigerator at 4 °C for two days. The seeds were directly sown in pots in a controlled environmental chamber in a short-day (8 h light/16 h dark) environment. Controlled chamber temperatures during the light and dark periods were 22 °C and 20 °C, respectively. Incandescent bulbs capable of emitting 160 µmol m^−2^ s^−2^ were used in the growth chambers, with a relative humidity of approximately 55%. A dome was placed over the flat until seeds began germination. After two weeks of growth, seedlings were transferred from the original pot into flats with moistened soil contained in 32 pots per flat (The Scotts Co., Marysville, OH, USA). Each Arabidopsis seedling was allocated to one pot. The transferred plants were grown in the same growth chamber. Plants were watered weekly, with a supplement of nitrogen/phosphorus/potassium fertilizer. The plants were kept in the chambers until needed, and a mature rosette (stage 3.9) could be observed at 5 weeks [35].

### 4.2. Bacterial Growth

*Pst* DC3000, the model pathogen used for Arabidopsis SAR induction [36] was streaked on agar media plates made according to the King’s B media protocol (20 g Protease peptone No. 3, 1.5 g K_2_HPO_4_ (s), 0.75 g MgSO_4_ (s), 10 mL glycerol, 15 g agar, and deionized H_2_O for a total volume of 1 L). The King’s B media was autoclaved, and antibiotics rifampicin (25 mg/L) and kanamycin (50 mg/L) were added to the King’s B media once it cooled down. *Pst* was streaked on this medium and incubated for 3 days at 28 °C. For liquid King’s B media, the composition was the same as for the plates except without agar. One fresh colony was used to inoculate the liquid culture, which was shaken overnight at 28 °C.

### 4.3. Stomata Aperture Measurements

Five-week-old WT and *npr1-1* plants were used for stomatal movement analysis. Primary inoculation occurred via needleless syringe injection, where the plants were either primed with *Pst* DC3000 (OD_600_ = 0.02) or mock-treated with 10 mM MgCl_2_. At three days post-inoculation, the leaf opposite to the injected leaf was detached for secondary treatment. In the secondary treatment, the plants were either floated in 10 mM MgCl_2_ or in *Pst* DC3000 (OD_600_ = 0.02) solution in small Petri-dishes. Three leaves were used for each time point and secondary treatment group, and only one leaf was collected from each plant. Stomatal apertures were measured at three time points: 0 h, 1 h, and 3 h. After each time point, the leaves were collected and peeled twice using the tape-peel method [37]. The abaxial side of the leaf was then placed on a microscope slide, and images were collected using a Leica DM 6000 B microscope (Leica, Bualo Grove, IL, USA). A total of 150 stomata measurements were needed for each time point for each plant line. Stomatal apertures were measured using ImageJ software (https://imagej.net/, 28 April 2023).

### 4.4. Pst entry and Growth Assays

To measure how many bacteria entered the apoplast after 3 h, nine independent biological replicates of WT plants Col-0 and nine replicates of *npr1-1* were grown for 5 weeks and prime-treated via injection with either *Pst* DC3000 (OD_600_ = 0.02) or mock-treated with 10 mM MgCl_2_. Three days after the infection, the leaf opposite to the one infected was detached and floated in *Pst* (OD_600_ = 0.02) solution for both mock and primed plants. After three hours of floating in solution, the leaf was placed in Falcon tube with 0.02% Silwet [38], vortexed for 10 s, wrapped in aluminum foil, and taken to a Laminar flow hood. An autoclaved hole-puncher was used to obtain one disk from each leaf (0.5 cm diameter), and the disk was placed in 100 µL sterile H_2_O. The leaf disk was then ground using a plastic grinding tip, and 10 µL was collected to make a 1:1000 serial dilution. A total of 100 µL was collected from the dilution and plated on agar media containing rifampicin (25 mg/L) and kanamycin (50 mg/L). After 3 days of incubation at 25 °C, the colonies on the plate were counted.

*Pst* growth assay was used to determine how much bacteria grew in the apoplast bypassing the stomatal barrier. Nine independent replicates of WT and nine independent *npr1-1* replicates were grown for 5 weeks and either mock- or prime-treated as described above. After three days of treatment, an opposite leaf was infiltrated with *Pst* DC3000 (OD_600_= 0.02), and the plant was left in the growth chamber for three days. The opposite leaf was then detached and washed in 0.02% Silwet, and a disk was taken from each leaf to make a 1:1000 serial dilution and plate it on media. Colonies were counted to determine how many bacteria were able to grow in the apoplast.

### 4.5. Stomata Density Assay

Stomata density was measured every week from the moment the plant was 2 weeks old until 8 weeks old. Every week, three *npr1-1* and three WT plants were collected, and two leaves were removed from each independent plant. With a total of six leaves for each genotype, three were used for he abaxial peel, while the other three were used for the adaxial peel. For the abaxial peel, tape was placed on both surfaces of the leaf and then removed from the adaxial side. Another layer of tape was placed and removed again. The tape containing the abaxial side of the leaf was then put on a microscope slide and imaged using a Leica DM 6000 B microscope (Leica, Bualo Grove, IL, USA). For the adaxial peel, adhesive was pipetted thinly onto a microscope slide, and the leaf was placed with the adaxial side touching the adhesive. The leaf was left for 10 min, and then, using a scalpel, the surface of the leaf was peeled in order to leave the adaxial surface on the microscope slide. Stomata were imaged in the same way as above. Two images of each of the leaf quadrants were taken per leaf, and the number of stomata were counted manually.

### 4.6. DAB, NBT, and MDA Analyses

H_2_O_2_ was detected in situ in rosette leaves of WT and *npr1-1* mock and primed plants by staining with 3,3ʹ-diaminobenzidine (DAB) (Sigma-Aldrich, catalog number: D8001), and superoxide (O_2_^-^) was detected by nitrotetrazolium blue chloride (NBT) (Sigma-Aldrich, catalog number: N6639) staining using a previously described method [12]. According to this method, DAB is oxidized by H_2_O_2_ in the presence of peroxidases, generating a dark brown precipitate that can be used as a stain to detect H_2_O_2_ presence and distribution in plant cells. NBT reacts with endogenous O_2_- and forms a formazan compound that is reflected by the presence of a dark blue stain. DAB and NBT staining solutions were prepared fresh in amber-colored bottles as follows: For NBT staining solution, a 50 mM sodium phosphate buffer (pH 7.5) was prepared by mixing 16 mL of 1M sodium phosphate monobasic with 84 mL of 1M sodium phosphate dibasic. Then, 0.1g of NBT was dissolved in 50 mL of the 50 mM sodium phosphate buffer in an amber-colored bottle to make a 0.2% NBT staining solution. DAB staining solution was prepared by dissolving 50 mg of DAB in 50 mL of sterile distilled water, and pH was adjusted to 3.8 using 0.1 M HCl. Three fully expanded distal leaves were collected from three individual 5-week-old Arabidopsis plants for each treatment group (WT mock, WT primed, *npr1-1* mock, and *npr1-1* primed), placed in 15 mL tubes, and immersed in either DAB or NBT staining solution. Tubes were wrapped in aluminum foil and kept overnight at room temperature. The next day, the staining solution was poured off, and chlorophyll was removed from the leaves by boiling in absolute ethanol for 10 min with intermittent shaking. Leaves were transferred to paper towels saturated with 60% glycerol and photographed. The experiment was repeated twice with similar results.

MDA content was measured using fully expanded leaves harvested from control and primed plants. They were ground to a fine powder in liquid nitrogen, followed by adding 3 mL of 10% trichloroacetic acid (TCA) to 0.2 g leaf tissue powder and incubated at 4 °C overnight. After centrifugation at 10,000 g at 4 °C for 20 min, the supernatant was transferred to a new tube, and 2 mL 0.6% thiobarbituric acid was added. The mixture was vortexed thoroughly, heated in boiling water for 15 min, cooled to 4 °C immediately and centrifuged at 10,000× *g* at 4 °C for 10 min. The absorbance of the supernatant was detected at wavelengths of 532 and 450 nm. The MDA content (µmol) was calculated as 6.45 * OD532—0.56 * OD450.

### 4.7. Protein Extraction, Digestion, and LC-MS/MS

Three biological replicates of each sample type were prepared for the proteomic experiments. For mock and primed samples, distal leaves were collected and immediately frozen in liquid nitrogen. These leaves were 72 h after one local leaf of the plant was injected with either 10 mM MgCl_2_ (mock) or the *Pst* DC3000 at OD_600_ of 0.02 (primed) to reveal molecular mechanisms underlying the priming and response to pathogens. After mock and priming, the distal leaves were exposed to either control or *Pst* DC3000 at OD_600_ of 0.2. Proteins were extracted using a TCA method [39]. Briefly, 0.5 g of frozen leaf tissue was ground in liquid nitrogen and mixed for 1 h in a solution of 10% TCA and 0.07% 2-mercaptoethanol (BME)/acetone. After centrifugation, the pellet was resuspended in a solution of 0.07% 2-mercaptoethanol (BME)/acetone twice and then dried by evaporation. Protein was extracted from the dried pellet by incubation for 1 h at room temperature in a lysis buffer (7M urea, 2M thiourea, 5% CHAPS, 2 mM tributylphosphine). The clean supernatant was collected, and the amount of protein was measured by Bradford assay. For each sample, 100 µg protein was cleaned by methanol and chloroform to remove lipids, detergent, and other contaminants. Protein samples were then resuspended in 50 mM ammonium bicarbonate, reduced using 10 mM dithiothreitol (DTT) for 1 h at 22 °C, alkylated with 55 mM chloroacetamide for 1 h in darkness, and then digested with trypsin at pH 8 for 16 h. Proteins were concentrated and cleaned using ZipTip (P10 size, Millipore Corporation, Billerica, MA, USA). A total of 6 µg of protein from each sample was resuspended in sterile water with 0.1% formic acid and loaded on the liquid chromatography column. Leaf sample peptides were separated using an Easy-nLC nano-HPLC liquid chromatography (EASY-nLC 1200) with a Thermo Fisher C18 reverse phase HPLC column, and analyzed using a Thermo Scientific Orbitrap Fusion Tribrid, quadrupole-ion trap-orbitrap MS/MS system (Thermo Scientific, San Jose, CA, USA).

The bottom-up proteomics data acquisition was performed on the EASY-nLC 1200 connected to the Orbitrap Fusion equipped with a nano-electrospray source (Thermo Scientific, San Jose, CA, USA). The peptide samples were loaded to a C18 trapping column (75 μm i.d. × 2 cm, Acclaim PepMap^®^ 100 particles with 3 μm size and 100 Å pores) and then eluted using a C18 analytical column (75 μm i.d. × 25 cm, 2 μm particles with 100 Å pore size). The flow rate was set at 300 nL/minute with solvent A (0.1% formic acid in water) and solvent B (0.1% formic acid and 99.9% acetonitrile) as the mobile phases. Separation was conducted using the following gradient: 2% of B over 0–0.5 min; 2–35% of B over 0.5–45 min, 35–98% of B over 45–46 min, and isocratic at 98% of B over 46–59 min, and then from 98–2% of B from 59 to 60 min. The equilibration time at 2% B was 15 min. A full MS1 scan (m/z 350–2000) was performed on the Orbitrap with a resolution of 120,000 at m/z 400. The automatic gain control (AGC) target is 2e5, with 50 ms as the maximum injection time. Monoisotopic precursor selection (MIPS) was enforced to filter for peptides. Peptides bearing +2 to +6 charges were selected with an intensity threshold of 1e4. Dynamic exclusion of 15 s was used to prevent resampling the high abundance peptides. The top-speed method was used for data-dependent acquisition within a cycle of 3 s. The MS/MS was carried out in the ion trap, with a quadrupole isolation window of 1.3 Da. Fragmentation of the selected peptides using the collision-induced dissociation (CID) was carried out at 35% of normalized collision energy with 10 ms activation time. The MS2 spectra were detected in the linear ion trap with the AGC target as 1e4 and the maximum injection time as 35 ms.

### 4.8. Proteomics Data Analysis

Proteome Discoverer™ 2.5 (Thermo Fisher, Waltham, MA, USA) was used for protein identification by searching the raw LC-MS/MS files against the Uniprot *A. thaliana* database. To create the processing workflow for label-free quantification, node selections in the Proteome Discoverer™ were as follows: spectrum files RC node and Spectrum Selector node with default settings using precursor mass minimum of 300 Da and maximum of 5 kDa. For protein identification, the Minora Feature detection node and Sequest HT search engine were used with settings of a maximum of two missed cleavage sites for trypsin digestion, a cross-correlation (XCorr) absolute threshold of 0.4, and a fragment ion cutoff percentage of 0.1. Precursor mass tolerance was set to 10 ppm and fragment mass tolerance was set to 0.8 Da. Selected dynamic modifications included N-ethylmaleimide (+124.048 Da (C)), oxidation (+15.995 Da (M)), and phosphorylation (+79,966 (S, T, Y)). The percolator node for protein identification had the parameters of a strict target FDR of 0.01 and a relaxed target FDR of 0.05. Consensus workflow node selection was MSF Files, PSM Grouper, Peptide Validator, Peptide and Protein Filter, Protein Scorer, Protein Grouping, Feature Mapper, and Precursor Ions Quantifier. Peptides were filtered to include only those with high confidence of identification (1% FDR), and quantification of unique peptides was performed using total area sum intensities. Quantified intensities were exported into Microsoft Excel, and ratios were calculated from the median-normalized peak intensity values.

## 5. Conclusions

In this study, we explored the role of NPR1 in systemic acquired stomatal immunity using WT and *npr1-1* Arabidopsis subjected to mock and priming pre-treatments and subsequent *Pst* exposure. While NPR1 does not affect stomatal density, the *npr1-1* mutant does not display *Pst*-triggered stomatal closure in systemic leaves, and the reopening mechanism, presumably driven by the SCREW-NUT functions, remains active. Further examination of proteomic differences among genotypes and treatments, the effects of priming, and subsequent *Pst* response, led to the conclusion that in WT Arabidopsis, stomatal closure in systemic leaves occurs via an NPR1-dependent ROS burst. In the *npr1-1*, stomata in systemic leaves remain open, promoting an alternative response characterized by translation regulation. This research underscores the critical role of NPR1 in systemic acquired stomatal immunity, thus enhancing our understanding of plant defense mechanisms at the first line of defense—stomatal guard cells.

## Figures and Tables

**Table 1:** The most differential abundant proteins in WT and *npr1-1* mutant. The fold change (FC) represents the protein abundances of *npr1-1* divided by WT.

Locus ID	Protein Name	*p* Value	Log_2_FC
AT5G13650	Elongation factor family protein	0.04	−5.30
AT1G33140	Ribosomal protein L6 family	0.03	5.16
AT3G47800	Galactose mutarotase-like superfamily protein	0.01	−3.76
AT5G10470	Kinesin like for actin-based chloroplast movement 1	0.01	−3.70
AT1G09340	Chloroplast RNA binding	0.05	−3.21
AT5G14590	Isocitrate/isopropylmalate dehydrogenase family protein	0.05	−3.18
AT1G16720	High chlorophyll fluorescence phenotype 173	0.03	−3.01
AT5G64050	Glutamate tRNA synthetase	0.01	−3.01
AT3G23810	S-adenosyl-l-homocysteine hydrolase 2	0.00	−2.96
AT1G79720	Eukaryotic aspartyl protease family protein	0.03	−2.94
AT2G35040	AICARFT/IMPCHase bienzyme family protein	0.00	−2.80
AT1G71720	Nucleic acid-binding proteins superfamily	0.02	−2.77
AT3G46740	Translocon at the outer envelope of chloroplasts 75-III	0.04	−2.71
AT4G02930	GTP binding elongation factor Tu family protein	0.00	−2.70
AT3G13750	Beta galactosidase 1	0.04	−2.42
AT3G18190	TCP-1/cpn60 chaperonin family protein	0.03	−2.41
AT3G07810	RNA-binding (RRM/RBD/RNP motifs) family protein	0.04	2.40
AT1G30530	UDP-glucosyl transferase 78D1	0.02	−2.35
AT5G60600	4-hydroxy-3-methylbut-2-enyl diphosphate synthase	0.03	−2.28
AT1G67700	Unknown protein	0.04	−2.22

**Table 2:** Proteins changed abundance significantly after 3 h *Pst* DC3000 infection in systemic leaves of the WT mock, WT primed, *npr1-1* mock, and *npr1-1* primed groups. The arrows indicate the change in protein abundance in 3 h *Pst* DC3000 infection compared to control (no *Pst* treatment).

Locus ID	Protein Name	WT Mock	WT Primed	*npr1-1* Mock	*npr1-1* Primed
AT1G66250	O-Glycosyl hydrolases family 17 protein	↑	↑	↑	↑
AT5G48540	Receptor-like protein kinase-related	↑	↑	↑	↑
AT5G52310	Low-temperature-responsive protein 78	↑	↑	↑	↑
AT3G44860	Farnesoic acid carboxyl-O-methyltransferase	↑	↑	↑	↑
AT5G03630	pyridine nucleotide-disulphide oxidoreductase	↑	↑	↑	↑
AT5G37360	Unknown protein	↑	↑	↑	↑
AT5G14040	Phosphate transporter 3;1	↑	↑	↑	↑
ATCG00020	Photosystem II reaction center protein A	↑	↑	↑	↑
AT5G36700	2-phosphoglycolate phosphatase 1	↑	↑	↑	↑
AT2G34930	Disease resistance / LRR family protein	↑	↑	↑	↑
AT1G53280	Class I glutamine amidotransferase-like	↑	↑	↑	↑
AT2G21170	Triosephosphate isomerase	↑	↑	↑	↑
AT3G12780	Phosphoglycerate kinase 1	↑	↑	↑	↑
AT5G19760	Mitochondrial substrate carrier	↑	↑	↑	↑
AT2G38270	CAX-interacting protein 2 (CXIP2)	↑	↑	↓	↓
AT5G58250	Unknown protein	↓	↓	↓	↓
AT1G15820	Light harvesting complex PS II subunit 6	↓	↓	↓	↓
AT4G25050	Acyl carrier protein 4	↓	↓	↓	↓
AT5G42980	Thioredoxin 3	↓	↓	↓	↓
AT1G08520	ALBINA 1	↓	↓	↓	↓
AT1G35680	Ribosomal protein L21	↓	↓	↓	↓

**Table 3:** The top 20 most significantly changed abundant proteins in *npr1-1* primed Arabidopsis after 3 h of pathogen infection (in the *npr1-1* primed group only).

Locus ID	Protein Name	*p*-Value	Log_2_FC
AT4G12830	Alpha/beta-Hydrolases (ABH) superfamily protein	0.01	−6.47
AT3G09630	Ribosomal protein L4/L1 family	0.01	6.07
AT5G54600	Translation protein SH3-like family protein	0.02	5.72
AT2G39460	Ribosomal protein L23AA	0.03	5.08
AT3G49010	60S ribosomal protein L13	0.02	5.00
AT1G20450	Dehydrin family protein	0.04	4.57
AT1G74970	Ribosomal protein S9	0.03	4.50
AT5G07090	Ribosomal protein S4 (RPS4A) family protein	0.03	4.28
AT1G23290	Ribosomal protein L18e/L15 superfamily protein	0.01	4.24
AT4G31700	Ribosomal protein S6	0.03	4.02
AT3G60770	Ribosomal protein S13/S15	0.02	3.95
AT4G35090	Catalase 2	0.02	3.94
AT2G40510	Ribosomal protein S26e family protein	0.01	3.80
AT2G43030	Ribosomal protein L3 family protein	0.02	3.73
AT3G02080	Ribosomal protein S19e family protein	0.01	3.60
AT1G26910	Ribosomal protein L16p/L10e family protein	0.01	3.42
AT5G15200	Ribosomal protein S4	0.01	3.28
AT4G18100	Ribosomal protein L32e	0.03	3.13
AT3G58510	DEA(D/H)-box RNA helicase family protein	0.04	−3.03
AT3G04550	Unknown protein	0.01	−2.93

## Data Availability

The raw MS-MS data for proteomic analysis can be found in the MassIVE database with accession number: MSV000091818.

## Supplementary Materials

The following supporting information can be downloaded at: https://www.mdpi.com/article/10.3390/plants12112137/s1, Table S1: Detailed MS and annotation information of proteins identified in this study. Figure S1: PCA of proteomic differences among the samples. (A) Result of proteomic differences among all the samples. (B) Results of WT samples (mock, primed, control and *Pst*). (C) Result of *npr1-1* samples (mock, primed, control and *Pst*). (D) Results of samples (WT and *npr1-1*) (mock, primed) without subsequent *Pst* exposure (control). (E) Results of samples (WT and *npr1-1*) (mock, primed) after subsequent *Pst* exposure.
