# Supplementary figures and images for: Role of NPR1 in Systemic Acquired Stomatal Immunity
Source: Plants (Basel). 2023 May 29;12(11):2137. doi: 10.3390/plants12112137 (PMC10255907; doi:10.3390/plants12112137)

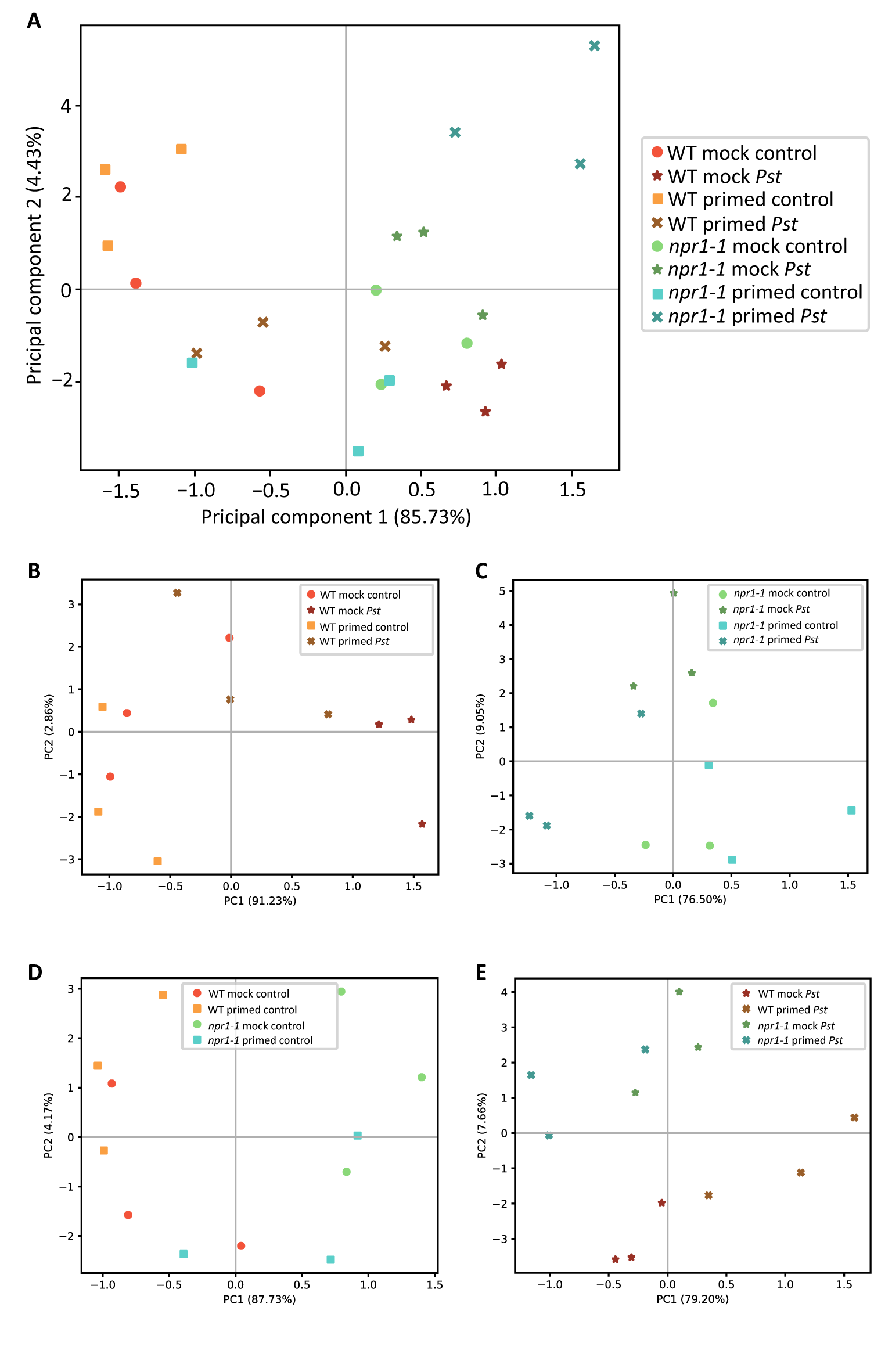

Supplement: Supplementary file 1 [file plants-12-02137-s001.zip › Figure S1.tif]
